# A case series: Four cases of urothelial carcinoma with sarcomatoid differentiation

**DOI:** 10.3389/fonc.2026.1782041

**Published:** 2026-04-15

**Authors:** Haosong Ma, Quanlv Xu, Guorun Zi, Donglin He, Changxing Ke

**Affiliations:** Department of Urology, The Second Affiliated Hospital of Kunming Medical University, Kunming, China

**Keywords:** differential diagnosis, immunotherapy, sarcomatoid differentiation, surgical resection, upper tract urothelial carcinoma

## Abstract

Urothelial carcinoma with sarcomatoid differentiation (UCSD) is a rare and highly aggressive variant of urothelial carcinoma associated with poor prognosis. UCSD originating in the upper urinary tract is extremely uncommon, and its variable morphological presentation, along with the lack of established treatment guidelines, often poses diagnostic and therapeutic challenges. We herein report four cases of upper tract UCSD treated with radical nephroureterectomy. All tumors exhibited varying proportions (40%-90%) of sarcomatoid components with diverse histological patterns. Postoperatively, patients received adjuvant chemotherapy. Through this case report and review of the relevant literature, we aim to enhance the understanding of this rare entity and contribute to the discussion on optimal treatment strategies for upper tract UCSD.

## Introduction

Urothelial carcinoma with sarcomatoid differentiation (UCSD), a rare (0.3%-0.6%) and highly aggressive morphologic pattern of urothelial carcinoma (UC), is characterized by a biphasic morphology of malignant epithelial and spindle-cell components and portends a significantly worse prognosis than conventional UC, with 5-year cancer-specific survival rates of only 37%-69.1% (1–4). According to the 2022 WHO Classification of Urinary and Male Genital Tumors (5th edition), “histologic subtypes” is now the preferred terminology over “variants” for distinct morphologies of urothelial carcinoma (5). UCSD is recognized as a distinct morphologic pattern rather than a separate entity, emphasizing that it represents a form of divergent differentiation along the epithelial-mesenchymal transition spectrum (6). The terms “sarcomatoid urothelial carcinoma” and “urothelial carcinoma with sarcomatoid differentiation” are used synonymously in the literature, with the latter being recommended to highlight its nature as a morphologic pattern of conventional urothelial carcinoma (7).Radical surgery is the cornerstone for localized disease, yet UCSD responds poorly to conventional platinum-based chemotherapy, posing a major therapeutic challenge (8). Biomarker-directed therapies have emerged as a potential strategy, though data on molecular profiling in UCSD remain extremely limited (9). This report presents a series of four upper tract UCSD cases. By integrating detailed clinicopathological features, molecular profiles, and outcomes following individualized adjuvant therapy, alongside a pertinent literature review, we aim to systematically delineate the characteristics of UCSD and share our experience in managing this refractory disease.

## Case reports

Immunohistochemical (IHC) staining was interpreted as follows: For diagnostic markers (CK7, GATA3, etc.), “(+)” indicates distinct staining in ≥10% of tumor cells; “weak+” indicates faint staining or positivity in <10% of cells. Her-2 was scored 0–3+ per 2018 ASCO/CAP guidelines. Ki-67 index is the percentage of positive nuclei in hotspots.

### Case 1

**Figure 1:**
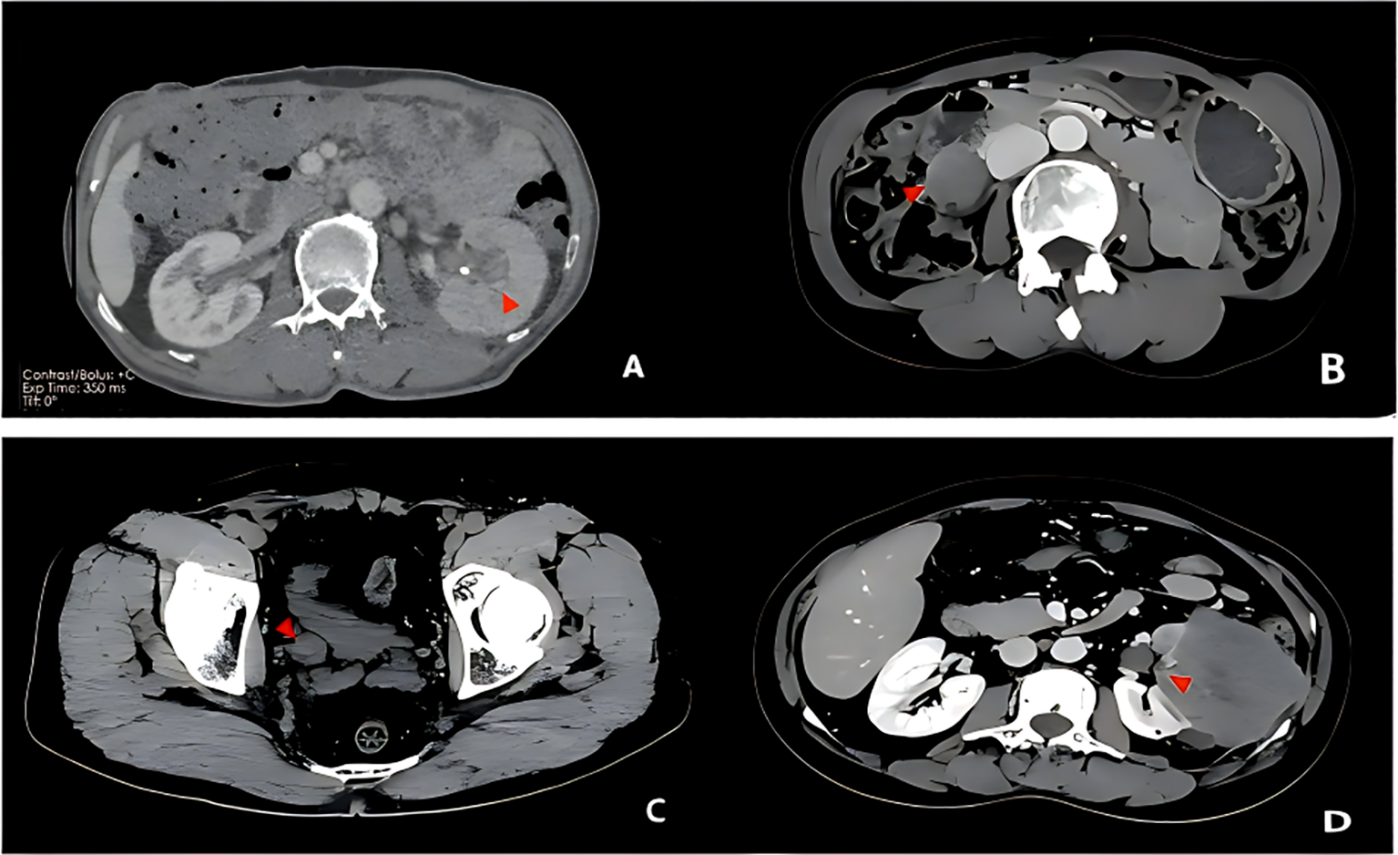
Enhanced CT images of Case 1 **(A)**, Case 2 **(B)**, Case 3 **(C)**, and Case 4 **(D)**.

**Figure 2:**
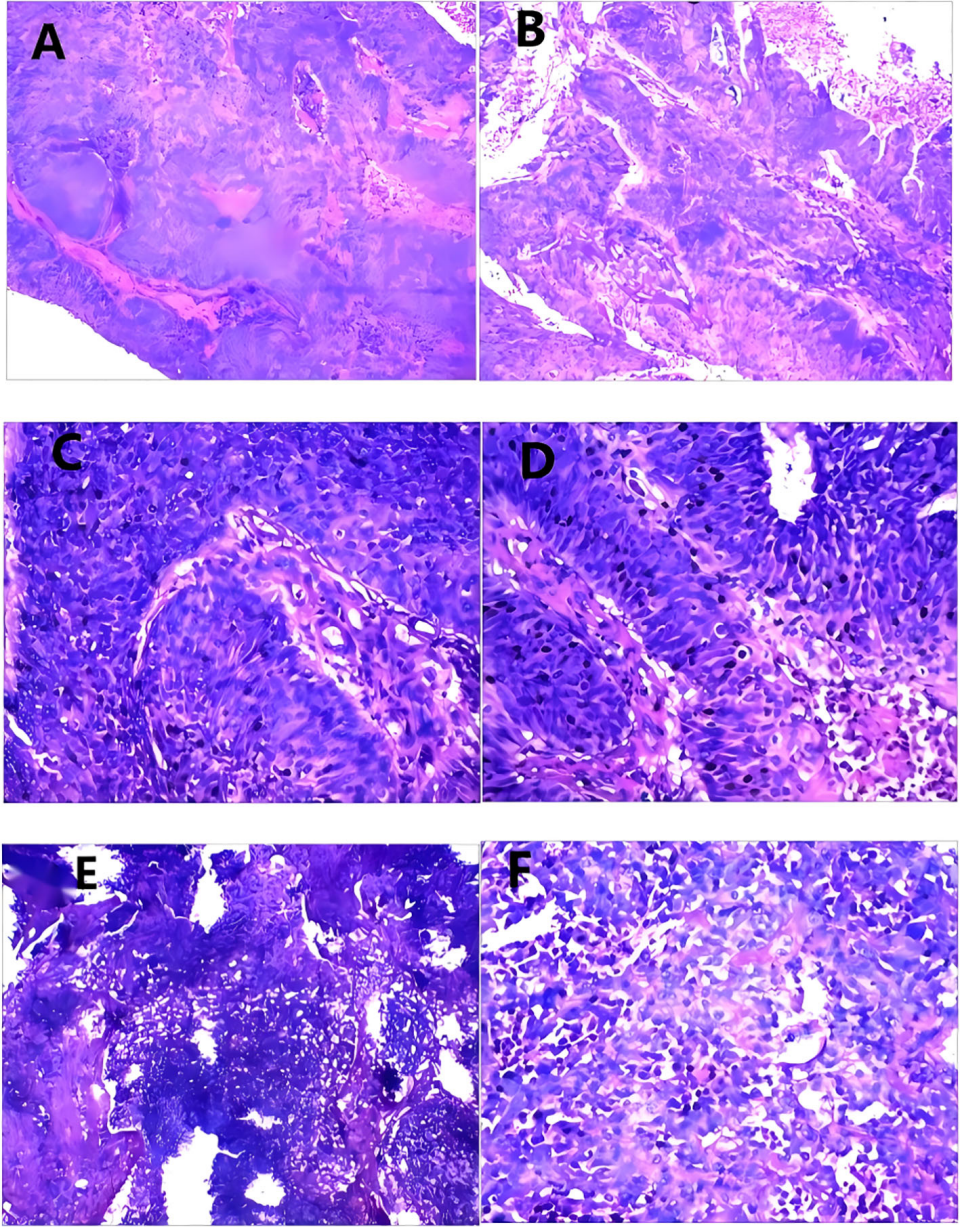
**(A, B)** Under low magnification, cancerous tissue infiltrated the renal parenchyma; no renal sinus fat was observed; and invasion of the ureteropelvic junction and venous end was also observed. **(C, D)** Under high magnification, focal lesions were observed with glandular differentiation, sarcomatoid differentiation, and osteoclast-type giant cell differentiation, with focal necrosis. **(E)** Under low magnification, Metastasis was observed in the hilar lymph nodes. **(F)** Under high magnification, Metastasis was observed in the hilar lymph nodes.

A 66-year-old male was admitted due to “recurrent painless gross hematuria for over 2 years, with recent aggravation for more than 10 days.” The patient experienced intermittent hematuria without obvious cause 2 years prior, with small blood clots and necrotic tissue in the urine. Ten days before admission, hematuria recurred, accompanied by persistent dull pain in the mid-upper abdomen. Abdominal CT upon admission suggested left hydronephrosis with patchy soft tissue shadows, indicating a possible mass (**Figure 1A**). The patient had a history of diabetes mellitus with fair glycemic control. Attempted ureteroscopy for diagnosis failed due to ureteral stenosis. Urine cytology from renal pelvis washing revealed atypical urothelial cells, suggestive of neoplastic lesions. After multidisciplinary discussion and thorough communication with the patient and family, radical surgery was decided. The patient underwent laparoscopic left nephroureterectomy with hilar lymph node dissection under general anesthesia. A cauliflower-like mass was observed in the left renal pelvis intraoperatively. Postoperative pathological examination and immunohistochemistry showed that the tumor cells were CK7 (+), CK5/6 (+), GATA3 (+), P63 (+), Urop-III (+), Her-2 (3+), Ki-67 (50%), P53 (+), and CK20 (-); the PD-L1 combined positive score (CPS) was 30. Microscopic observation revealed that the tumor exhibited biphasic differentiation, with a mixed distribution of epithelioid and spindle-shaped sarcomatoid regions, as well as scattered multinucleated osteoclast-like giant cells (**Figures 2A–D**). The diagnosis was high-grade urothelial carcinoma of the left renal pelvis, with a differentiated degree, including glandular, sarcomatoid (approximately 40%), and osteoclast-like giant cell components. The tumor infiltrated the renal parenchyma, with one metastasis (pT3N1Mx) in one of the six hilar lymph nodes (**Figures 2E, F**). The sarcomatoid component accounted for approximately 40% of the tumor volume. MMR status was not assessed due to insufficient tissue. Postoperatively, the patient received four cycles of tislelizumab combined with gemcitabine and cisplatin, which were well tolerated. However, during the fifth cycle at approximately 5 months post-surgery, he developed severe myelosuppression requiring isolation and intensive supportive care. The sixth cycle at approximately 8 months post-surgery was complicated by leukopenia which resolved after treatment. At approximately 9 months post-surgery, he underwent transurethral resection of bladder tumor. Subsequently, he received two cycles of nanoparticle albumin-bound paclitaxel combined with camrelizumab at approximately 10 and 11 months post-surgery. At 11 months post-surgery, follow-up imaging revealed a ground-glass nodule in the right upper lobe, multiple solid nodules in the right middle and lower lobes (suspicious for pulmonary metastases), and a mass in the posterior bladder wall (suspicious for bladder cancer). The patient was admitted for his ninth cycle of chemotherapy at approximately 12 months post-surgery. Despite recommendations to modify the treatment regimen due to disease progression, the patient declined. He was discharged and was alive with disease progression at the last follow-up.

### Case 2

**Figure 3:**
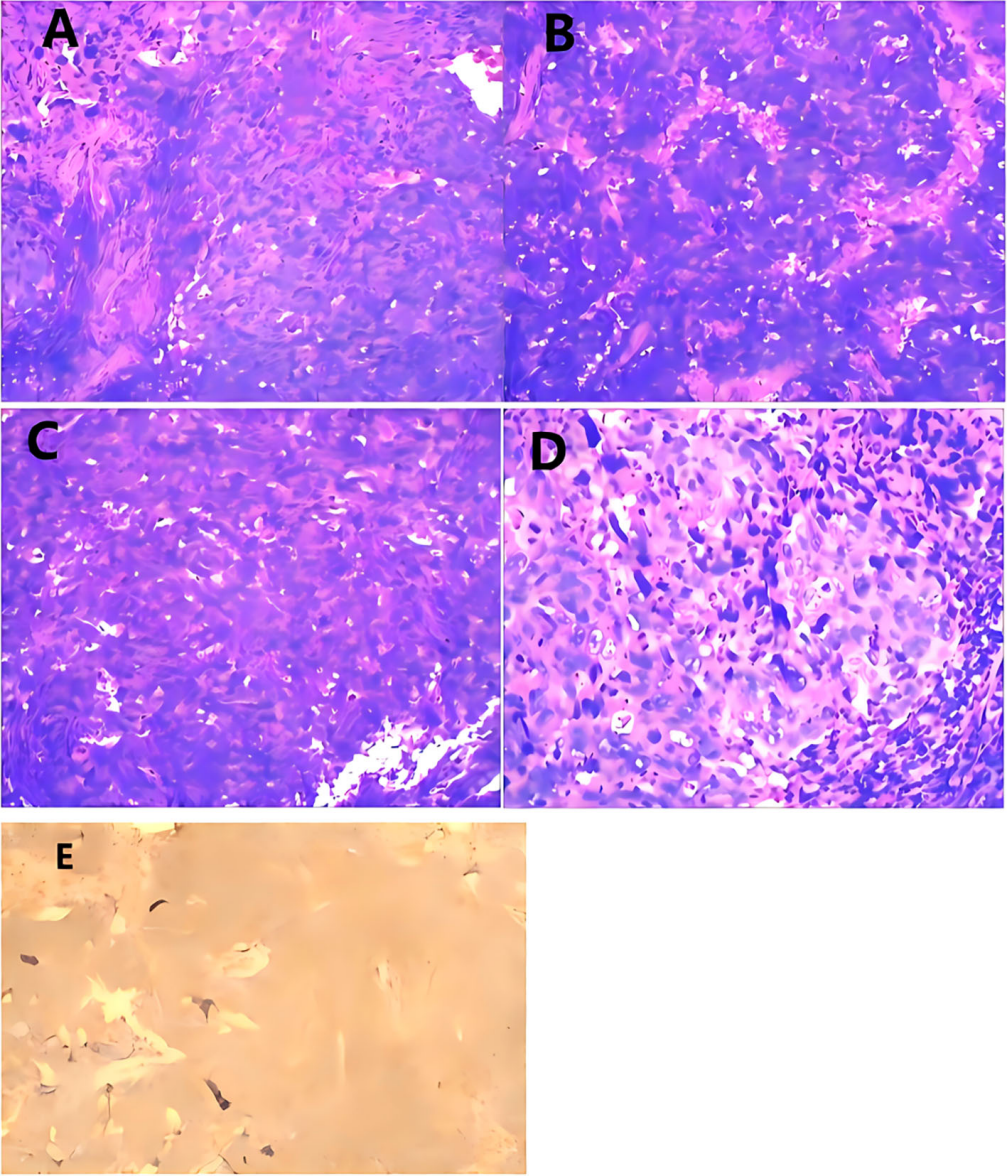
**(A, B)** Under low magnification, Tumor infiltrates to the muscularis propria. **(C, D)** Under high magnification, observed with sarcomatoid differentiation, and osteoclast-type giant cell differentiation. **(E)** VIM(+).

A 55-year-old female was admitted due to “painless gross hematuria for 1 month, aggravated for 2 weeks.” The patient experienced intermittent total gross hematuria without obvious cause one month prior, worsening over the two weeks before admission. CT urography (CTU) revealed a mass at the right pelvi-ureteric junction, highly suggestive of urothelial carcinoma, accompanied by moderate hydronephrosis and hydroureter of the right upper tract (**Figure 1B**). MRI further confirmed the mass at the right ureteropelvic junction. Renal dynamic imaging showed moderately impaired right renal function with a glomerular filtration rate (GFR) of 13.52 mL/min. The patient had a history of hysterectomy and appendectomy. After multidisciplinary discussion and thorough communication with the patient and family, radical surgery was decided. The patient underwent laparoscopic right nephroureterectomy with bladder cuff excision under general anesthesia. Postoperative pathology showed: high-grade invasive urothelial carcinoma with sarcomatoid differentiation (UCSD) of the right ureter, with tumor invasion into the muscularis propria (pT2N0M0). Microscopically, the tumor exhibited a mixture of high-grade urothelial carcinoma components and markedly atypical spindle sarcomatoid cells (**Figures 3A–D**). The sarcomatoid component accounted for approximately 60% of the tumor volume. Immunohistochemistry showed tumor cells positive for CK7(+), VIM(+)(**Figure 3E**), GATA3(focally weak+), Ki-67(up to 90%), P53(+), Her-2(1+), and negative for CK20(-); mismatch repair proteins were intact (pMMR) with normal expression of MLH1, MSH2, MSH6, and PMS2; PD-L1 Combined Positive Score (CPS) was 90.The postoperative course was complicated by transient intestinal obstruction and moderate anemia, which resolved with conservative management. At approximately 5 weeks post-surgery, the patient initiated adjuvant therapy with toripalimab combined with disitamab vedotin, adjusted for her creatinine clearance of 50 mL/min. At the last follow-up (18 months post-surgery), the patient remained alive with no evidence of tumor recurrence on surveillance imaging and tolerated treatment well.

### Case 3

**Figure 4:**
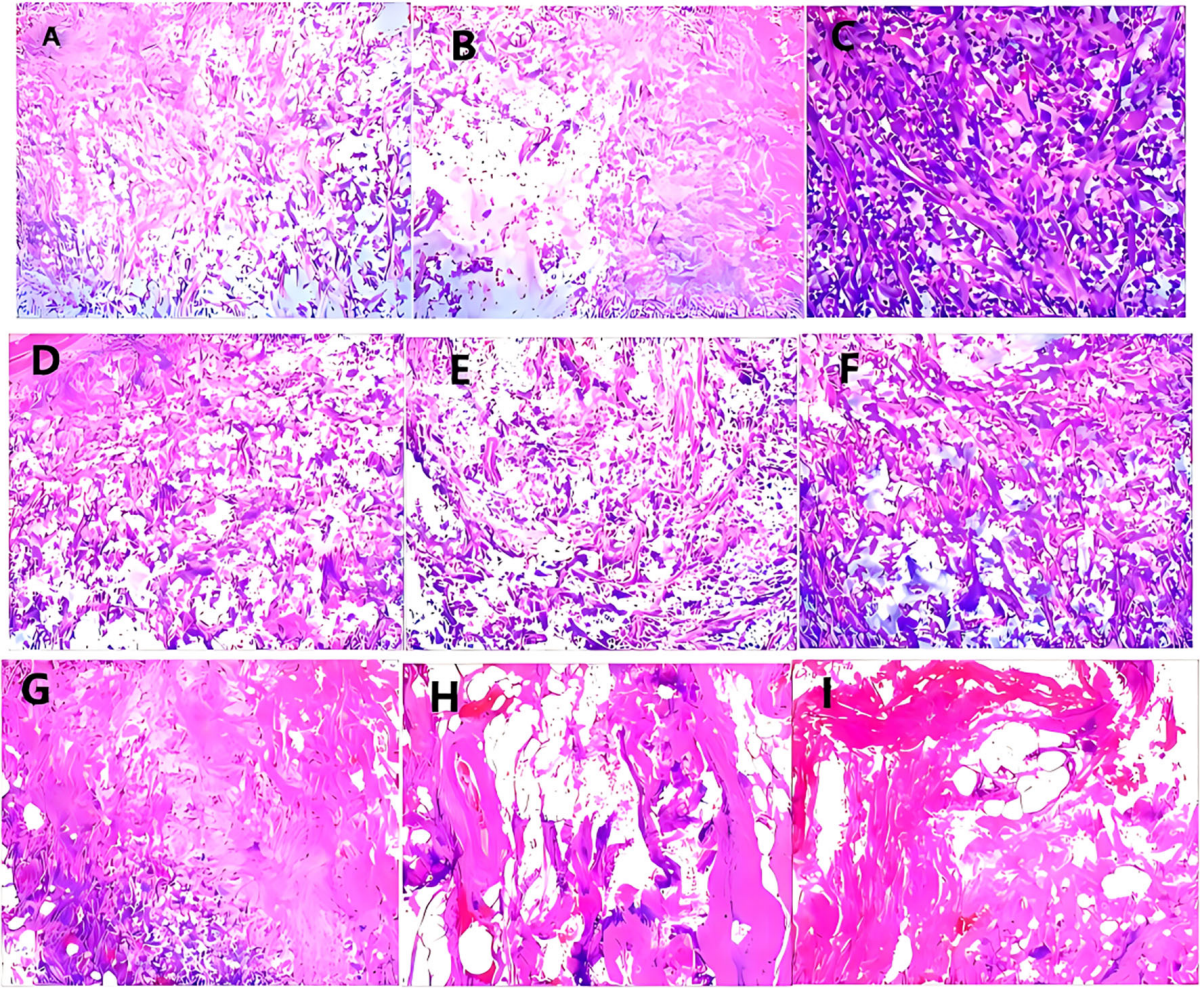
**(A)** Under low magnification, sarcomatous urothelial carcinoma was observed infiltrating the renal parenchyma. **(B)** Under medium magnification, sarcomatous urothelial carcinoma was observed infiltrating the renal parenchyma. **(C)** Under high magnification, sarcomatous urothelial carcinoma was observed infiltrating the renal parenchyma. **(D)** Under low magnification, the tumor infiltrated the entire ureteral wall and extended into the surrounding adipose tissue, accompanied by multiple intravascular tumor thrombi. **(E)** Under medium magnification, the tumor infiltrated the entire ureteral wall and extended into the surrounding adipose tissue, accompanied by multiple intravascular tumor thrombi. **(F)** Under high magnification, the tumor infiltrated the entire ureteral wall and extended into the surrounding adipose tissue, accompanied by multiple intravascular tumor thrombi. **(G)** Under low magnification, sarcomatous urothelial carcinoma infiltrates within fibroadipose tissue. **(H)** Under medium magnification, sarcomatous urothelial carcinoma infiltrates within fibroadipose tissue. **(I)** Under high magnification, sarcomatous urothelial carcinoma infiltrates within fibroadipose tissue.

The patient was a 69-year-old male admitted to the hospital due to right lower abdominal pain for more than one month. Preoperative urine cytology revealed suspicious malignant tumor cells, suggestive of urothelial carcinoma. Pelvic CT scan showed thickening and enhancement of the wall of the lower right ureter near the bladder inlet, suggestive of urothelial carcinoma, accompanied by upstream ureteral dilation and hydronephrosis (**Figure 1C**). Right ureteral biopsy showed chronic mucosal inflammation with scattered atypical urothelial cells, highly suggestive of high-grade urothelial carcinoma, which was subsequently supported by immunohistochemical staining. After multidisciplinary discussion, the patient underwent laparoscopic right nephroureterectomy under general anesthesia. Postoperative pathology confirmed sarcomatoid urothelial carcinoma involving the right kidney and ureter. Tumor infiltration was identified in the renal parenchyma and periureteral adipose tissue (**Figures 4A-C**). Within the ureter, the tumor infiltrated through the full thickness of the ureteral wall into the surrounding adipose tissue (**Figures 4D–F**). Multiple intravascular tumor thrombi were observed (**Figures 4G-I**). The bladder wall resection margin was negative for tumor. Immunohistochemistry showed tumor cells positive for VIM, GATA3 (partial), Ki-67 (70%), P53 (focal), and CK7 (partial), and negative for Her-2, P40, P63, CK5/6, and CK20. Mismatch repair proteins MSH6, MSH2, MLH1, and PMS-2 were intact, indicating microsatellite stability (MSS). PD-L1 Combined Positive Score (CPS) was 90. Microscopically, the tumor exhibited prominent sarcomatoid features with sheets of atypical spindle cells. The patient recovered smoothly in the early postoperative period and was discharged stably. However, approximately two months postoperatively, rapid disease progression occurred, accompanied by extensive local recurrence and distant metastasis. Postoperative imaging follow-up two months later revealed a large pelvic mass invading the bladder, bilateral seminal vesicles, prostate, and right obturator internus muscle, with suspected rectal involvement; multiple enlarged lymph nodes in the retroperitoneum and pelvis, suggestive of metastasis; concurrent tumor-related small bowel obstruction, multiple pulmonary nodules, and bilateral pleural effusion. Laboratory tests showed elevated tumor markers, including NSE 48.67 ng/mL, SCCA 2.98 ng/mL, and ferritin 845.61 ng/mL. Renal function progressively deteriorated, with creatinine reaching a peak of 757 μmol/L, and an estimated glomerular filtration rate decreasing to 6 mL/min, accompanied by severe anemia (hemoglobin 64 g/L). Given the rapid disease progression and critical clinical status, including malignant bowel obstruction, gastrointestinal bleeding, acute kidney injury requiring hemodialysis, and severe cancer pain, the patient was no longer eligible for systemic chemotherapy or immunotherapy. The patient received comprehensive supportive care, including nasoenteric catheter placement, percutaneous nephrostomy, blood transfusions, analgesia, and dialysis. Due to rapid disease progression and extremely poor overall physical condition, no adjuvant anti-tumor therapy was administered. The patient’s family requested discharge, and the patient signed discharge papers under end-of-life care. Approximately three months post-surgery, the patient passed away due to disease progression.

### Case 4

**Figure 5:**
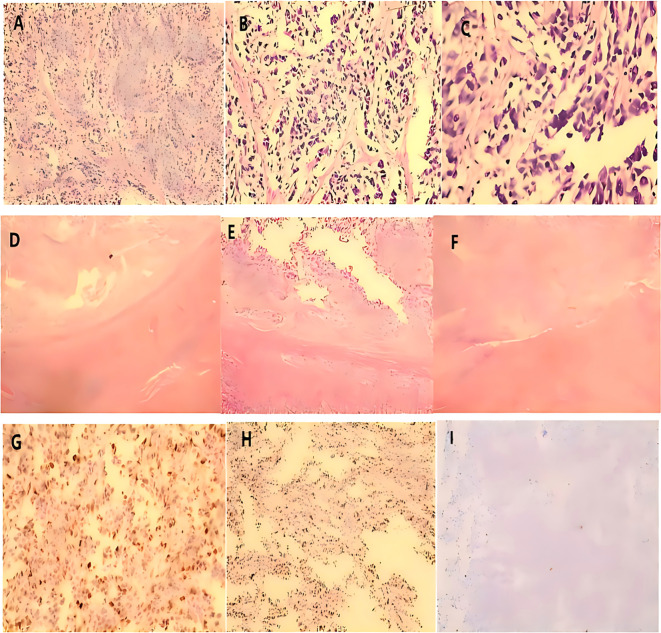
**(A)** Under low magnification, sarcomatous urothelial carcinoma was observed infiltrating the renal parenchyma. **(B)** Under medium magnification, sarcomatous urothelial carcinoma was observed infiltrating the renal parenchyma. **(C)** Under high magnification, sarcomatous urothelial carcinoma was observed infiltrating the renal parenchyma. **(D)** Under low magnification, sarcomatoid urothelial carcinoma invades local tissues of the spleen. **(E)** Under medium magnification, sarcomatoid urothelial carcinoma invades local tissues of the spleen. **(F)** Under high magnification, sarcomatoid urothelial carcinoma invades local tissues of the spleen. **(G)** GATA3(+). **(H)** Ki-67(weak+). **(I)** PD-L1(-).

A 52-year-old female patient was admitted to the hospital with left upper quadrant abdominal pain for two weeks. Enhanced CT revealed an irregular exogenous soft tissue mass of approximately 11.3 × 9.6 cm within the left kidney, invading the lower pole of the spleen (**Figure 1D**). Multiple cystic lesions were also observed within the left kidney. After multidisciplinary discussion and thorough communication with the patient and her family regarding the risk of intestinal invasion, preoperative bowel preparation was performed, and radical surgery was decided upon. On August 29, 2025, the patient underwent laparoscopic left nephrectomy combined with total splenectomy under general anesthesia. Postoperative pathology confirmed invasive sarcomatoid urothelial carcinoma involving the left kidney. Postoperative pathology confirmed sarcomatoid urothelial carcinoma involving the left kidney, with tumor infiltrating the renal parenchyma, perirenal adipose tissue (**Figures 5A–C**), and spleen parenchyma (**Figures 5D–F**). Immunohistochemistry showed that tumor cells were VIM(+), GATA3 (partially+) (**Figure 5G**), Ki-67 (70%) (**Figure 5H**), CKL(+), CKH(+), CD56 (partially+), CK7(-), CK20(-), P40(-), P63 (weakly+ in a few cells), PAX-8(-), CD10(-), and Her-2(0). Mismatch repair proteins MLH1, MSH2, MSH6, and PMS-2 were all positively expressed, indicating intact mismatch repair function (pMMR) and consistent with microsatellite stability (MSS). The PD-L1 combined positive score (CPS) was <1 (**Figure 5I**). No metastasis was found in the renal hilar lymph nodes. The final pathological stage was pT4N0Mx. The patient recovered well after surgery and began adjuvant therapy with toripalimab combined with veentumumab about 4 months after surgery. At the 8-month follow-up, the patient continued to receive regular chemotherapy and immunotherapy, which was well tolerated.

A summary of the clinical timeline for all four cases is presented in **Table 1**.

**Table 1:** Clinical timeline of four upper tract UCSD cases.

Event	Case 1	Case 2	Case 3	Case 4
Symptom onset	2 years prior	1 month prior	1 month prior	2 weeks prior
Diagnosis method	Imaging and urine cytology at admission	CTU at admission	Preoperative biopsy	Imaging at admission
Surgery	Laparoscopic left nephroureterectomy	Laparoscopic right nephroureterectomy	Laparoscopic right nephroureterectomy	Laparoscopic left nephrectomy + splenectomy
Adjuvant therapy start	Post-op week 6	Post-op week 5	Not initiated	Post-op month 4
Disease progression	Post-op month 11	None	Post-op month 2	None
Last follow-up	Post-op month 12	Post-op month 18	Post-op month 3	Post-op month 8
Status at last follow-up	Alive with disease	No evidence of disease	Died of disease	No evidence of disease

*UCSD, urothelial carcinoma with sarcomatoid differentiation; CTU, computed tomography urography; Post-op, postoperative.*

### Review of related literature

The following section presents a narrative review of the literature on urothelial carcinoma with sarcomatoid differentiation, summarizing current evidence on clinical features, imaging, pathology, molecular advances, and treatment.

### Clinical features

Urothelial carcinoma with sarcomatoid differentiation (UCSD) is a rare and highly aggressive morphologic pattern of bladder cancer, accounting for approximately 0.3%–0.6% of all bladder cancers (2, 10). It shows a distinct demographic preference, predominantly affecting elderly males, with a median age at diagnosis between 66 and 72 years and a male-to-female ratio of about 3:1 (2, 3). Smoking is a well-established major risk factor. The most common clinical presentation is painless gross hematuria, which may be accompanied by lower urinary tract symptoms such as dysuria, frequency, and urgency (11). Due to its inherently high aggressive biological behavior, most patients present with muscle-invasive (≥pT2) or even locally advanced disease at initial diagnosis, often with lymph node or distant metastasis (4, 12). Our case series aligns with this, as all patients were diagnosed at pT2 stage or higher, with Case 1 showing lymph node metastasis. The prognosis of UCSD is extremely poor and significantly worse than that of conventional UC. Reported 5-year cancer-specific survival (CSS) rates range from 37% to 69.1%, with a median overall survival (OS) of approximately 14 to 22.5 months (3, 4, 12). Regarding treatment, radical surgery is the cornerstone for localized disease. However, UCSD responds poorly to platinum-based neoadjuvant or adjuvant chemotherapy, which has not demonstrated a clear survival benefit (8, 13). Consequently, clinical management is highly challenging, necessitating the exploration of new effective therapeutic strategies.

### Imaging features

While imaging findings of UCSD are not pathognomonic, multimodal imaging can reveal its aggressive features and aid in clinical staging. Ultrasound typically shows a solid, broad-based mass within the bladder lumen or upper urinary tract, with heterogeneous echotexture and abundant blood flow signals. Non-contrast computed tomography (CT) demonstrates an irregular soft-tissue density mass, often with necrosis and cystic changes. On dynamic contrast-enhanced CT, all four cases in our series demonstrated heterogeneous arterial-phase hyperenhancement, followed by rapid washout in the delayed phase. This enhancement pattern is distinct from that of sarcomatoid carcinoma of the liver, which typically shows reduced arterial enhancement with delayed-phase progressive enhancement (14). The observed hyperenhancement in UCSD likely reflects underlying tumor angiogenesis. This is consistent with histopathological observations that sarcomatoid areas exhibit significantly richer tumor stroma infiltration, a microenvironment that necessitates active neoangiogenesis to support tumor growth and progression (15). CT is the preferred method for assessing local invasion and distant metastasis. Magnetic resonance imaging (MRI), with its superior soft tissue resolution, outperforms CT in accurately evaluating tumor invasion depth (e.g., distinguishing T2 from T3 stages) and invasion of surrounding organs. On positron emission tomography-computed tomography (PET-CT), UCSD exhibits high metabolic activity, with significantly increased fluorodeoxyglucose (FDG) uptake in primary and metastatic sites, with maximum standardized uptake values (SUVmax) potentially as high as 22.1 (10). This is valuable for detecting occult metastases and comprehensive staging (10). Regarding the mechanisms underlying high FDG uptake, evidence suggests that the sarcomatoid component may show disproportionately higher FDG avidity than the epithelial component. This is likely attributable to its higher proliferative activity (as reflected by Ki-67 indices of 70-90% in our series) and a metabolically active tumor microenvironment characterized by abundant tumor-associated macrophages (15). This differential metabolic activity has practical implications for biopsy targeting, as FDG-avid areas are more likely to yield diagnostic sarcomatoid tissue. The CT findings in our series (e.g., heterogeneous enhancement, invasion of surrounding structures) are consistent with these aggressive imaging features.

### Pathological features

Pathological diagnosis of SUC integrates gross, histological, and immunohistochemical findings. Grossly, tumors are typically large (avg. 4–6 cm), polypoid or nodular with a broad base, and the cut surface frequently shows extensive hemorrhage, necrosis, and cystic degeneration (6). Histologically, the defining biphasic morphology comprises malignant epithelial components (usually high-grade urothelial carcinoma, sometimes with squamous or glandular features) admixed with malignant mesenchymal-like (sarcomatoid) areas. The sarcomatoid component most often presents as a high-grade spindle cell sarcoma; special morphological variants include myxoid change or heterologous differentiation, such as osteosarcoma or chondrosarcoma (6, 16). Our cases reflect this spectrum: Case 1 displayed sarcomatoid areas with osteoclast-like giant cells, Cases 2 and 3 were predominantly composed of sarcomatoid spindle cells, and Case 4 was a massive, sarcomatoid-dominant neoplasm. Immunohistochemically, the epithelial component is strongly positive for Pan-CK, CK7, CK20, EMA, and p63, while GATA-3 expression is variable (~16–44%) and often focal or weak (15), as seen in our Cases 2–4. The sarcomatoid component is consistently vimentin-positive (~100%), and the critical diagnostic feature is the frequent co-expression of cytokeratins (notably CK AE1/AE3) within these spindle cells, which distinguishes SUC from pure sarcomas (7)—a pattern confirmed in all our cases where sarcomatoid areas co-expressed epithelial markers (CK7, CKL, CKH) and vimentin. Notably, sarcomatoid areas show significantly higher infiltration of CD163+ M2-type tumor-associated macrophages compared to epithelial regions (15). The Ki-67 proliferation index is typically very high (>60–70%), underscoring the tumor’s aggressive biology (10), which aligns with the 50–90% Ki-67 indices in our series. A key diagnostic pitfall is the limited tissue from transurethral or ureteroscopic biopsies, which can lead to sampling error and misdiagnosis (17), exemplified by the initial non-diagnostic biopsy in Case 1 and highlighting the need for deep sampling and evaluation of radical specimens. Beyond tumor stage, the proportion of the sarcomatoid component (>50%) is an important independent prognostic factor associated with worse outcomes (3).In our series, the proportion of sarcomatoid differentiation ranged from 40% to approximately 90%, with three of four cases exceeding the 50% threshold (**Table 2**).

**Table 2:** Clinicopathological features and treatment outcomes of four UCSD patients.

Feature	Case 1	Case 2	Case 3	Case 4
Age/Sex	66/M	55/F	69/M	52/F
Sarcomatoid component	40%	60%	~80-90%	~70%
Pathological stage	pT3N1Mx	pT2N0M0	pT3N0Mx	pT4N0Mx
Key IHC Profile	CK7(+), CK5/6(+), GATA3(+), P63(+), Urop-III(+), P53(+)	CK7(+), VIM(+), GATA3(focal weak+), P53(+)	VIM(+), GATA3(partial+), CK7(partial+), P53(focal+), CK20(-), P40(-)	VIM(+), GATA3(partial+), CKL(+), CKH(+), CD56(partial+), CK7(-), CK20(-), P40(-), P63(weak+ in few cells), PAX-8(-), CD10(-)
Ki-67 index	50%	90%	70%	70%
PD-L1 expression	CPS: 30	CPS: 90	CPS: 90	CPS: <1
HER2 status	3+	1+	Negative	0
MMR status	Not assessed	pMMR	MSS	pMMR
Adjuvant therapy	Tislelizumab + GC → Nab-paclitaxel + Camrelizumab	Toripalimab + DV	None	Toripalimab + DV
Follow-up (months)	12 (AWD)	18 (NED)	3 (DOD)	8 (NED)

M, male; F, female; GC, gemcitabine + cisplatin; DV, disitamab vedotin; AWD, alive with disease; NED, no evidence of disease; DOD, died of disease; CPS, Combined Positive Score; pMMR, proficient mismatch repair; MSS, microsatellite stable. IHC definitions: “(+)” = staining in ≥10% of tumor cells; Ki-67 = % positive nuclei in hotspots; PD-L1 CPS = PD-L1+ cells per 100 tumor cells. For Key IHC Profile, markers are listed with their expression patterns;.

### Advances in molecular understanding

Recent years have seen growing insights into the pathogenesis of SUC. SUC is considered the “final common pathway” of urothelial carcinoma dedifferentiation through epithelial-mesenchymal transition (EMT) (18). EMT confers migratory, invasive, and stem cell-like properties to cancer cells. Utilizing digital spatial analysis technology, it has been found that SUC areas exhibit significantly richer tumor stroma infiltration, predominantly composed of CD163+ M2 macrophages and activated fibroblasts, which play immunosuppressive and tumor-promoting roles (15). Gene expression levels of transforming growth factor-beta (TGF-β) are significantly upregulated in SUC areas. TGF-β is a core cytokine driving EMT, creating a microenvironmental loop that promotes tumor invasion and immune evasion (15). This may partially explain the observed high PD-L1 expression in our case series.

### Genomic features of sarcomatoid urothelial carcinoma

Although genomic profiling was not performed in our cases, recent studies have elucidated key genomic features of SUC. Compared to conventional urothelial carcinoma, SUC exhibits a distinct genomic landscape characterized by a higher tumor mutational burden (TMB) and frequent alterations in TP53, RB1, and PIK3CA (15). Mutations in chromatin remodeling genes, including ARID1A and KMT2D, are also commonly observed, reflecting global epigenetic dysregulation (15). The sarcomatoid component shows enrichment of epithelial-mesenchymal transition (EMT)-related gene signatures, with upregulation of TGF-β signaling and downregulation of cell adhesion molecules such as E-cadherin (encoded by CDH1) (6, 15). These genomic alterations contribute to the aggressive phenotype, chemoresistance, and potential vulnerability to immune checkpoint inhibitors. The high PD-L1 expression observed in three of our four cases (CPS 30-90) is consistent with reports linking SUC to an immunosuppressive tumor microenvironment driven by these genomic changes (15).

### Immune microenvironment and therapeutic implications

The enrichment of M2-type tumor-associated macrophages and the potential role of CD8+ T-cell infiltration in SUC have important therapeutic implications. Previous studies have demonstrated that sarcomatoid areas show significantly higher infiltration of CD163+ M2 macrophages compared to epithelial regions, contributing to an immunosuppressive microenvironment that may facilitate immune evasion (15). While we were unable to assess macrophage polarization or CD8+ T-cell infiltration in our cases due to the retrospective nature of this series, these immune components represent important areas for future investigation. The heterogeneous treatment responses observed in our patients—particularly the rapid progression of Case 3 despite high PD-L1 expression—suggest that a more comprehensive immune profiling, including assessment of tumor-infiltrating lymphocytes and myeloid-derived suppressor cells, may be necessary to predict response to immune checkpoint blockade in UCSD. A critical consideration when interpreting PD-L1 expression in UCSD is the substantial methodological heterogeneity inherent to PD-L1 immunohistochemical assessment. As reviewed by Sanguedolce et al., PD-L1 testing in urothelial carcinoma is characterized by marked variability across antibody clones (e.g., 22C3, SP142, SP263) and scoring systems (CPS vs. TPS), with interobserver variability further complicating result interpretation (19, 20). These challenges are particularly pronounced in upper tract disease and rare histological variants such as UCSD, where standardized protocols are lacking. Moreover, intratumoral heterogeneity—a well-recognized feature of UCSD given its biphasic morphology—adds another layer of complexity, as PD-L1 expression may differ substantially between epithelial and sarcomatoid components (20). Consequently, PD-L1 expression status should be interpreted with appropriate caution and, in the current state of evidence, should not serve as the sole determinant for immunotherapy decision-making in this rare entity.

### Differential diagnosis

Urothelial carcinoma with sarcomatoid differentiation (UCSD) requires meticulous differentiation from other spindle cell lesions of the upper urinary tract, relying on histomorphology combined with a systematic immunohistochemical (IHC) panel (7, 21). FIGURE 4 provides a diagnostic algorithm outlining the key steps.

The principal differential diagnoses include:

1. Sarcomatoid renal cell carcinoma (sRCC): This represents the most critical differential, particularly for renal parenchymal tumors. sRCC retains a renal phenotype, typically showing strong expression of renal lineage markers such as PAX8, PAX2, and CAIX, while urothelial markers (e.g., GATA3, p63) are generally negative (18).In our series, Case 4 was distinguished from sRCC based on PAX8/PAX2 negativity and focal GATA3 positivity.
2. Primary mesenchymal sarcomas: Examples include leiomyosarcoma and fibrosarcoma. These are inherently mesenchymal neoplasms and thus are cytokeratin (CK) negative, while expressing vimentin and lineage-specific markers (e.g., SMA, Desmin) (7).
3. Inflammatory myofibroblastic tumor (IMT): A potentially low-grade malignant tumor more common in younger patients, characterized by a proliferation of spindle-shaped myofibroblasts within an inflammatory background. Approximately 50% of cases express ALK, while CK is typically negative (22).
4. Sarcomatoid carcinoma from other origins or metastases: Invasion or metastasis from sarcomatoid carcinomas of the prostate, colorectum, or gynecological tract must be excluded through clinical context and specific markers (e.g., PSA, CDX2, PAX8/WT-1) (7).

Systematic Diagnostic Approach: For upper tract spindle cell lesions, a stepwise diagnostic strategy is recommended (21). Evaluation should begin with cytokeratin (CK) staining, such as CK7 or pan-CK, to confirm epithelial differentiation. If CK is positive, the next step is to determine the primary site using a combination of urothelial markers (GATA3, p63) and renal markers (PAX8, PAX2). Positivity for GATA3 and/or p63 supports UCSD, while PAX8 or PAX2 positivity indicates sarcomatoid renal cell carcinoma (sRCC) (18).If CK is negative, mesenchymal tumors are favored, prompting evaluation for ALK, SMA, desmin, and other lineage-specific markers to diagnose primary sarcomas or inflammatory myofibroblastic tumor (IMT) (7, 22).Applying this approach to our series, the diagnosis of UCSD was confirmed in all four cases by co-expression of CK and vimentin in the sarcomatoid spindle cells, along with at least focal GATA3 positivity. In Case 4, negative staining for PAX8 and PAX2 was critical in excluding sRCC despite the tumor’s large size and renal parenchymal invasion. In all cases, primary sarcomas were ruled out by the presence of CK expression in at least a subset of tumor cells. Regardless of the final diagnosis, assessment of PD-L1 (reporting both CPS and TPS is advised) and mismatch repair (MMR) protein status is strongly recommended for therapeutic guidance.

### Treatment and prognosis

Owing to its rarity, no standard treatment for sarcomatoid urothelial carcinoma (SUC) exists, and management generally follows principles for muscle-invasive urothelial carcinoma guided by retrospective evidence (8). Radical nephroureterectomy with lymph node dissection remains the cornerstone for localized disease (10, 23), yet SUC demonstrates poor sensitivity to platinum-based chemotherapy, with no clear survival benefit from neoadjuvant or adjuvant approaches (8, 13). Prognosis is poor, with 5-year cancer-specific survival of 37%-69.1% (3, 4) and median overall survival of 14-22.5 months (3, 10), influenced by advanced stage (≥pT3), lymph node involvement, and sarcomatoid component >50% (3, 5). In our series, all four patients underwent radical nephroureterectomy with postoperative adjuvant therapy individualized based on molecular profiles: Case 1 (pT3N1Mx, PD-L1 CPS 30, HER2 3+) received tislelizumab plus gemcitabine-cisplatin, later switched to nab-paclitaxel plus camrelizumab due to progression; Case 2 (pT2N0M0, PD-L1 CPS 90, HER2 1+) received toripalimab plus disitamab vedotin and remained disease-free at 18 months; Case 3 (pT3N0Mx, PD-L1 CPS 90, HER2 negative) experienced rapid postoperative progression, precluding systemic therapy, and died at 3 months; Case 4 (pT4N0Mx, PD-L1 CPS <1, HER2 0) received toripalimab plus disitamab vedotin and remained disease-free at 8 months. High PD-L1 expression (CPS 30-90) was observed in three cases, consistent with reports linking SUC to an immunosuppressive microenvironment (15), and while responses to immune checkpoint inhibitors in SUC are heterogeneous (24), two of three patients who received timely biomarker-guided adjuvant therapy achieved short-term disease-free survival. Case 3 illustrates that high PD-L1 expression alone does not guarantee favorable outcomes in the setting of rapid progression, and we recommend simultaneous reporting of CPS and TPS in SUC, as CPS may underestimate PD-L1 expression in tumors with sparse lymphocytic infiltration.

### Patient perspective

Upon diagnosis of this rare and aggressive cancer, all patients experienced significant anxiety. The multidisciplinary explanation that their postoperative treatment would be guided by personalized PD-L1 testing—rather than a standard approach—provided substantial reassurance and hope. Patients have tolerated immunotherapy well and express profound relief at remaining disease-free during follow-up. Written informed consent has been obtained from all participants for publication of their anonymized details and this perspective.

### Limitations

This study is a retrospective case series with a small sample size, reflecting the rarity of UCSD. PD-L1 testing utilized different antibody clones and scoring systems (CPS vs. TPS) across cases, highlighting the need for standardized protocols. MMR status was not assessed in Case 1 due to insufficient tissue, and genomic profiling was not performed in any case. Additionally, immunohistochemical staining for immune cell subsets, including CD8+ T cells and tumor-associated macrophages (e.g., CD163, CD68), was not performed, limiting our ability to characterize the immune microenvironment and its correlation with treatment response. Although two patients showed favorable short-term responses to immunotherapy, the rapid progression and death of Case 3 despite high PD-L1 expression underscores heterogeneous treatment outcomes. Longer follow-up is warranted to assess durability of response and late toxicities.

## Conclusion

We report four cases of upper tract urothelial carcinoma with sarcomatoid differentiation (UCSD). All patients underwent radical nephroureterectomy followed by adjuvant therapy guided by molecular profiles, including PD-L1 expression, HER2 status, and mismatch repair status. Three of four cases exhibited high PD-L1 expression (CPS 30–90), while one case showed low expression (CPS <1). With follow-up periods ranging from 3 to 18 months, two patients remained disease-free (Case 2 at 18 months, Case 4 at 8 months), one patient was alive with disease progression (Case 1 at 12 months), and one patient died of disease three months post-surgery (Case 3), reflecting the heterogeneous and often aggressive clinical course of UCSD. Integrated with literature analysis, this series confirms that UCSD is a rare and aggressive morphologic pattern of urothelial carcinoma with a poor prognosis. Diagnosis requires recognition of biphasic histology and systematic immunohistochemical evaluation to exclude mimics, particularly sarcomatoid renal cell carcinoma (sRCC). Key markers include CK, GATA3, p63, Vimentin, PAX8, and PAX2.The high PD-L1 expression observed in three cases aligns with recent evidence linking UCSD to an immunosuppressive tumor microenvironment and epithelial-mesenchymal transition (EMT) process (15). Although genomic profiling was not performed in this series, published studies have identified frequent alterations in TP53, RB1, and PIK3CA, as well as enrichment of EMT-related gene signatures in UCSD (6, 15).We recommend incorporating PD-L1 testing (reporting both CPS and TPS) and MMR status assessment into routine pathological evaluation of UCSD to guide immunotherapy decisions. The favorable outcomes in two of our patients provide preliminary clinical evidence supporting postoperative adjuvant immunotherapy for UCSD with high PD-L1 expression. However, the rapid progression in Case 3 despite high PD-L1 expression underscores that this biomarker alone is insufficient to predict response. Future multicenter studies with integrated genomic and immune profiling are needed to identify robust predictive biomarkers and optimize treatment strategies for this challenging disease.

## Data Availability

The original contributions presented in the study are included in the article/supplementary material. Further inquiries can be directed to the corresponding author/s.
